# DECOLONIZING DISSEMINATION OF RESEARCH ENSURES LOCAL RELEVANCE AND SUSTAINABILITY OF RESEARCH BEYOND THE GRANT

**DOI:** 10.1093/geroni/igae098.1471

**Published:** 2024-12-31

**Authors:** Jordan Lewis, Steffi Kim

**Affiliations:** University of Alaska Fairbanks, College of Indigenous Studies, Fairbanks, Alaska, United States; University of Alaska, Anchorage, Alaska, United States

## Abstract

Engaging in community-based participatory research with Indigenous communities has become an accepted approach in both qualitative and quantitative research methods, as well as by funders. Our team has been conducting community-engaged research with Alaska Native communities for over 16-years. We have conducted qualitative, in-depth, interviews with 162 Elders representing 21 participating communities to explore the concept of successful aging and the role of their community in the aging process. Alaska Native successful aging consists of six elements of successful aging, or “Eldership:” emotional wellbeing, spirituality, community engagement, Native ways of life, generativity, and physical health. These elements serve as the foundation of how communities define who is an Elder and what is important when considering who has aged successfully or not. This presentation will briefly discuss challenges of community engagement, but focus on the successes of disseminating the findings back to the community using locally driven recommendations. Western forms of study dissemination products tend to be publications and presentations, which support the researchers’ goals and objectives, but do not always translate to community interests and ideas. Our team has been successful in developing culturally safe and innovative dissemination products, including berry buckets, coffee mugs, and sweatshirts. We will discuss how to navigate funding guidelines, how to develop to products in collaboration with community members and Elders, as well as the share the impacts these products have on making the data accessible to the Elders and the community, and not just the academic community and funder.

